# 4-Acetamido­anilinium nitrate monohydrate

**DOI:** 10.1107/S1600536812019393

**Published:** 2012-05-05

**Authors:** Sana Riahi, Mohamed Lahbib Mrad, Valeria Ferretti, Frederic Lefebvre, Cherif Ben Nasr

**Affiliations:** a Laboratoire de Chimie des Matériaux, Faculté des Sciences de Bizerte, 7021 Zarzouna, Tunisia; b Dipartimento di Chimica and Centro di Strutturistica Diffrattometrica, University of Ferrara, via L. Borsari 46, I-44121 Ferrara, Italy; c Laboratoire de Chimie Organometallique de Surface, 43 Bd du 11 Novembre 1918, 69616 Villeurbanne Cedex, France

## Abstract

In the title hydrated salt, C_8_H_11_N_2_O^+^·NO_3_^−^·H_2_O, the N—C bond distances [1.349 (2) and 1.413 (2) Å] along with the sum of the angles (359.88°) around the acetamide N atom clearly indicate that the heteroatom has an *sp* ^2^ character. The ammonium group is involved in a total of three N—H⋯O hydrogen bonds, two of these are with a water mol­ecule, which forms two O—H⋯O hydrogen bonds. All these hydrogen bonds link the ionic units and the water mol­ecule into infinite planar layers parallel to (100). The remaining two N—H⋯O inter­actions in which the ammoniun group is involved link these layers into an infinite three-dimensional network.

## Related literature

For the structural diversity of amine salts, see: Tooke *et al.* (2004 ▶). For related nitrate compounds, see: Dai & Chen (2011 ▶); Pourayoubi *et al.* (2011 ▶); Berrah *et al.* (2011 ▶). For hydrogen-bond patterns in related compounds, see: Flores-Alamo *et al.* (2010 ▶). For details of graph-set theory, see: Bernstein *et al.* (1995 ▶).
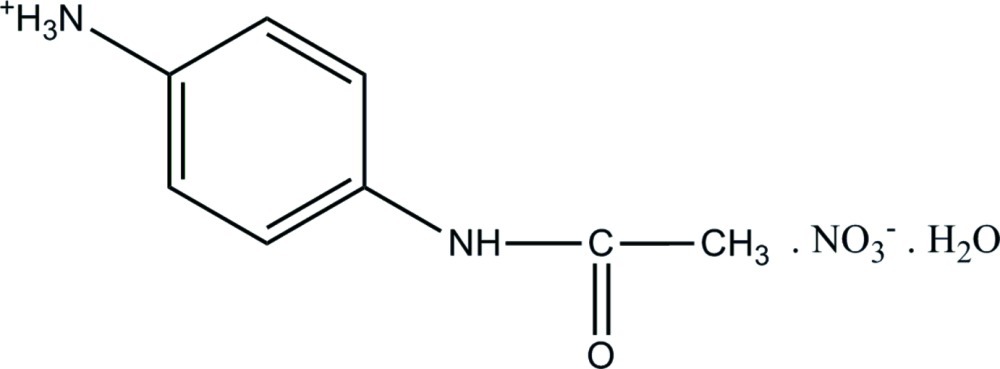


## Experimental

### 

#### Crystal data

C_8_H_11_N_2_O^+^·NO_3_^−^·H_2_O
*M* *_r_* = 231.21
Monoclinic, 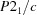

*a* = 4.1059 (1) Å
*b* = 23.1112 (5) Å
*c* = 11.4702 (3) Å
β = 92.942 (1)°
*V* = 1087.00 (5) Å^3^
*Z* = 4
Mo *K*α radiation
μ = 0.12 mm^−1^
*T* = 295 K
0.41 × 0.32 × 0.25 mm

#### Data collection

Nonius KappaCCD diffractometer
4741 measured reflections
2606 independent reflections
1885 reflections with *I* > 2σ(*I*)
*R*_int_ = 0.022

#### Refinement

*R*[*F* ^2^ > 2σ(*F* ^2^)] = 0.046
*wR*(*F* ^2^) = 0.142
*S* = 1.06
2606 reflections
197 parameters
All H-atom parameters refined
Δρ_max_ = 0.24 e Å^−3^
Δρ_min_ = −0.19 e Å^−3^

### 

Data collection: *COLLECT* (Nonius, 1997 ▶); cell refinement: *DENZO-SMN* (Otwinowski & Minor, 1997 ▶); data reduction: *DENZO-SMN*; program(s) used to solve structure: *SIR97* (Altomare *et al.*, 1999 ▶); program(s) used to refine structure: *SHELXL97* (Sheldrick, 2008 ▶); molecular graphics: *ORTEPIII* (Burnett & Johnson, 1996 ▶); software used to prepare material for publication: *SHELXL97* and *WinGX* (Farrugia, 1999 ▶).

## Supplementary Material

Crystal structure: contains datablock(s) global, I. DOI: 10.1107/S1600536812019393/lr2060sup1.cif

Structure factors: contains datablock(s) I. DOI: 10.1107/S1600536812019393/lr2060Isup2.hkl

Supplementary material file. DOI: 10.1107/S1600536812019393/lr2060Isup3.cml

Additional supplementary materials:  crystallographic information; 3D view; checkCIF report

## Figures and Tables

**Table 1:** Hydrogen-bond geometry (Å, °)

*D*—H⋯*A*	*D*—H	H⋯*A*	*D*⋯*A*	*D*—H⋯*A*
O1*W*—H1*W*⋯O1	0.93 (3)	1.79 (3)	2.689 (2)	161 (3)
O1*W*—H2*W*⋯O3	0.78 (3)	2.03 (3)	2.805 (2)	172 (3)
N1—H3⋯O2^i^	0.91 (2)	2.24 (2)	3.117 (2)	161 (2)
N2—H4⋯O1*W*^ii^	0.93 (2)	1.87 (2)	2.796 (2)	173 (2)
N2—H5⋯O2^iii^	0.89 (3)	2.14 (3)	3.008 (2)	165 (3)
N2—H6⋯O1*W*^iv^	0.93 (3)	1.95 (2)	2.827 (2)	156 (2)

Symmetry codes: (i) 
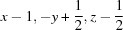
; (ii) 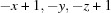; (iii) 
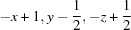
; (iv) 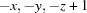.

**Fig. 1.**
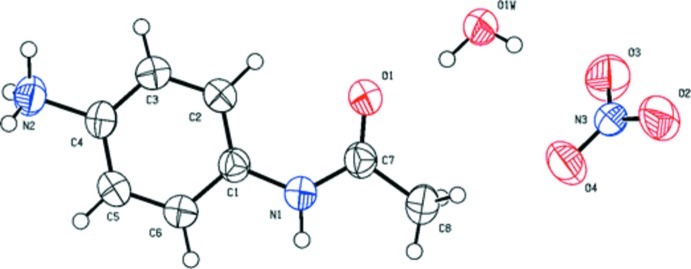
A view of (I) showing 50% probability displacement ellipsoids.

**Fig. 2.**
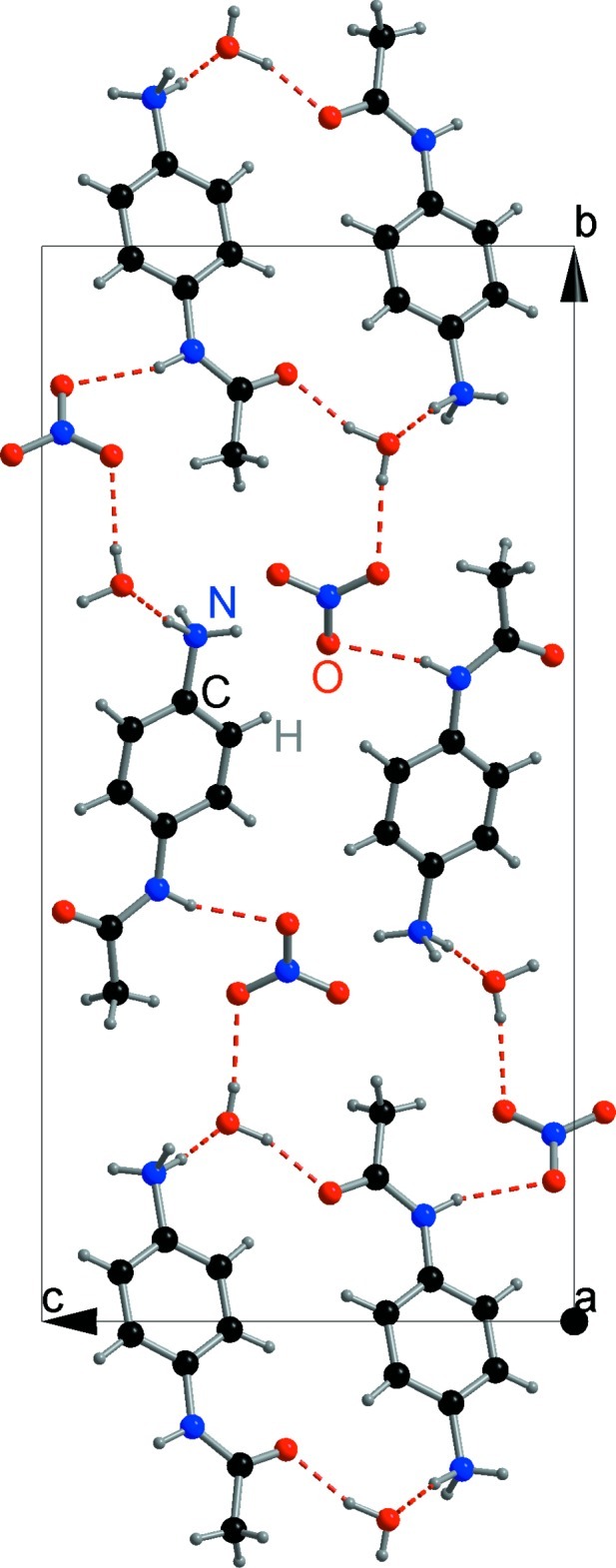
A view, down the a axis, showing a layer of the structure. The hydrogen bonding transforming the layers in a three-dimenional network has been omitted for clarity.

## supplementary crystallographic information

### Comment

Salts of amine show interesting structural diversity governed mainly by hydrogen bonds (Tooke *et al.*, 2004), whose crystal pattern seems to be strongly influenced by the nature of the anion (Flores-Alamo *et al.*, 2010). We report here the crystal structure of one such compound, 4-acetamidoanilinium nitrate monohydrate, (I), formed from the reaction of 4-acetamidoaniline and nitric acid. The asymmetric unit of the title salt, shown in Fig. 1, contains one protonated 4-acetamidoanilinium cation, one nitrate anion and one water molecule. The NO_3_^-^ anion geometry agrees with that observed in similar compounds (Dai & Chen, 2011; Pourayoubi *et al.*, 2011; Berrah *et al.*, 2011). In the organic cation the bond length distance N1—C7 = 1.349 (2) Å and the sum of the angles around N1 atoms = 359.88 ° clearly indicate that the heteroatom N1 has a *sp*^2^ character. The N2 nitrogen atom is involved in a positive charge-assisted N—H···O hydrogen bond with a neighboring water molecule (N2···O1w = 2.796 (2) Å). Moreover, the water molecule forms two O—H···O interactions (O1W···O1 = 2.689 (2) Å and O1W···O3 = 2.805 (2) Å) with, respectively, the O1 oxygen atom belonging to the carbonyl group and the O3 atom of the adjacent NO_3_^-^ anion. The weak hydrogen bond N1—H3···O2 (N1···O2 = 3.117 (2) Å) contributes to the robustness of the crystal architecture but it is not influencing the overall crystal packing pattern (Table 1, Fig. 2). Within the structure, the graph-set motif (Bernstein *et al.*, 1995) *R*_4_^4^(22) is formed by two cations and two water molecules, which in turn link cations and anions to form infinite planar layers parallel to (1 0 0) (Fig. 2). These layers are interconnected via further hydrogen bonds to form a three dimensional network.

### Experimental

Commercial 4-acetamidobenzenamine (3 mmol) was dissolved in water/HNO_3_ (50:1 *v*/*v*) solution. Colorless single crystals of the title compound, suitable for X-ray analysis, were obtained after slowly evaporation of the solvent at room temperature.

### Refinement

All H atoms were located in successive difference Fourier maps and refined riding on its parents atoms with U*iso*=1.2 and 1.5U*eq*(parent atom).

### Crystal data

**Table:**

C_8_H_11_N_2_O^+^·NO_3_^−^·H_2_O	*F*(000) = 488
*M**_r_* = 231.21	*D*_x_ = 1.413 Mg m^−^^3^
Monoclinic, *P*2_1_/*c*	Mo *K*α radiation, λ = 0.71073 Å
Hall symbol: -P 2ybc	Cell parameters from 4741 reflections
*a* = 4.1059 (1) Å	θ = 3.0–28.0°
*b* = 23.1112 (5) Å	µ = 0.12 mm^−^^1^
*c* = 11.4702 (3) Å	*T* = 295 K
β = 92.942 (1)°	Prismatic, pale yellow
*V* = 1087.00 (5) Å^3^	0.41 × 0.32 × 0.25 mm
*Z* = 4	

### Data collection

**Table:**

Nonius KappaCCD diffractometer	1885 reflections with *I* > 2σ(*I*)
Radiation source: fine-focus sealed tube	*R*_int_ = 0.022
Graphite monochromator	θ_max_ = 28.0°, θ_min_ = 3.7°
φ scans and ω scans	*h* = −5→5
4741 measured reflections	*k* = −30→27
2606 independent reflections	*l* = −15→15

### Refinement

**Table:**

Refinement on *F*^2^	Primary atom site location: structure-invariant direct methods
Least-squares matrix: full	Secondary atom site location: difference Fourier map
*R*[*F*^2^ > 2σ(*F*^2^)] = 0.046	Hydrogen site location: difference Fourier map
*wR*(*F*^2^) = 0.142	All H-atom parameters refined
*S* = 1.06	*w* = 1/[σ^2^(*F*_o_^2^) + (0.0773*P*)^2^ + 0.1041*P*] where *P* = (*F*_o_^2^ + 2*F*_c_^2^)/3
2606 reflections	(Δ/σ)_max_ < 0.001
197 parameters	Δρ_max_ = 0.24 e Å^−^^3^
0 restraints	Δρ_min_ = −0.19 e Å^−^^3^

### Special details

**Table:**

Geometry. All e.s.d.'s (except the e.s.d. in the dihedral angle between two l.s. planes) are estimated using the full covariance matrix. The cell e.s.d.'s are taken into account individually in the estimation of e.s.d.'s in distances, angles and torsion angles; correlations between e.s.d.'s in cell parameters are only used when they are defined by crystal symmetry. An approximate (isotropic) treatment of cell e.s.d.'s is used for estimating e.s.d.'s involving l.s. planes.
Refinement. Refinement of *F*^2^ against ALL reflections. The weighted *R*-factor *wR* and goodness of fit *S* are based on *F*^2^, conventional *R*-factors *R* are based on *F*, with *F* set to zero for negative *F*^2^. The threshold expression of *F*^2^ > σ(*F*^2^) is used only for calculating *R*-factors(gt) *etc*. and is not relevant to the choice of reflections for refinement. *R*-factors based on *F*^2^ are statistically about twice as large as those based on *F*, and *R*- factors based on ALL data will be even larger.

### Fractional atomic coordinates and isotropic or equivalent isotropic displacement parameters (Å^2^)

**Table:**

	*x*	*y*	*z*	*U*_iso_*/*U*_eq_	
O1	0.1624 (3)	0.11922 (5)	0.45863 (11)	0.0662 (4)	
O2	0.6017 (4)	0.36949 (6)	0.53827 (14)	0.0878 (5)	
O3	0.3474 (5)	0.30471 (6)	0.63123 (12)	0.0866 (5)	
O4	0.3293 (4)	0.30565 (6)	0.44397 (12)	0.0766 (4)	
O1W	0.2790 (3)	0.18436 (6)	0.65055 (11)	0.0570 (3)	
N1	−0.0860 (3)	0.09798 (5)	0.28298 (12)	0.0500 (3)	
N2	0.2062 (4)	−0.13752 (6)	0.21089 (16)	0.0543 (4)	
N3	0.4244 (3)	0.32626 (5)	0.53870 (12)	0.0515 (3)	
C1	0.0001 (3)	0.03922 (6)	0.27013 (12)	0.0431 (3)	
C2	0.1814 (4)	0.00752 (7)	0.35317 (14)	0.0515 (4)	
C3	0.2493 (4)	−0.05022 (7)	0.33325 (15)	0.0518 (4)	
C4	0.1367 (3)	−0.07607 (6)	0.23129 (13)	0.0447 (3)	
C5	−0.0431 (4)	−0.04540 (7)	0.14791 (15)	0.0524 (4)	
C6	−0.1123 (4)	0.01213 (7)	0.16734 (14)	0.0519 (4)	
C7	−0.0072 (4)	0.13406 (6)	0.37245 (13)	0.0480 (4)	
C8	−0.1396 (5)	0.19416 (8)	0.3608 (2)	0.0621 (5)	
H1	0.259 (4)	0.0238 (8)	0.4267 (17)	0.061 (5)*	
H2	0.369 (5)	−0.0731 (9)	0.3872 (18)	0.065 (5)*	
H3	−0.205 (5)	0.1131 (9)	0.2208 (18)	0.060 (5)*	
H4	0.374 (5)	−0.1507 (9)	0.2619 (18)	0.067 (5)*	
H5	0.243 (6)	−0.1417 (13)	0.135 (3)	0.103 (9)*	
H6	0.040 (6)	−0.1603 (12)	0.239 (2)	0.095 (8)*	
H7	−0.229 (4)	0.0345 (9)	0.1086 (19)	0.065 (5)*	
H8	−0.123 (5)	−0.0614 (9)	0.0796 (18)	0.064 (5)*	
H9	−0.251 (9)	0.2023 (17)	0.290 (4)	0.141 (12)*	
H10	0.017 (8)	0.2231 (14)	0.371 (3)	0.115 (9)*	
H11	−0.272 (8)	0.2008 (15)	0.415 (3)	0.124 (11)*	
H1W	0.219 (6)	0.1691 (11)	0.578 (2)	0.087 (7)*	
H2W	0.288 (6)	0.2175 (13)	0.639 (2)	0.088 (8)*	

### Atomic displacement parameters (Å^2^)

**Table:**

	*U*^11^	*U*^22^	*U*^33^	*U*^12^	*U*^13^	*U*^23^
O1	0.0952 (9)	0.0479 (7)	0.0534 (7)	0.0049 (6)	−0.0168 (6)	−0.0067 (5)
O2	0.1177 (12)	0.0606 (8)	0.0825 (10)	−0.0398 (8)	−0.0211 (8)	0.0080 (7)
O3	0.1328 (13)	0.0705 (9)	0.0578 (8)	−0.0060 (8)	0.0158 (8)	0.0140 (7)
O4	0.0939 (10)	0.0746 (9)	0.0600 (8)	−0.0196 (7)	−0.0086 (7)	−0.0111 (6)
O1W	0.0760 (8)	0.0460 (7)	0.0485 (7)	−0.0006 (5)	−0.0023 (5)	0.0004 (5)
N1	0.0605 (7)	0.0410 (7)	0.0475 (7)	0.0017 (5)	−0.0055 (6)	−0.0018 (5)
N2	0.0544 (8)	0.0424 (7)	0.0662 (10)	0.0028 (6)	0.0025 (7)	−0.0084 (6)
N3	0.0673 (8)	0.0381 (6)	0.0479 (7)	−0.0029 (5)	−0.0074 (6)	0.0047 (5)
C1	0.0473 (7)	0.0393 (7)	0.0428 (7)	−0.0037 (5)	0.0037 (6)	−0.0003 (6)
C2	0.0631 (9)	0.0466 (8)	0.0438 (8)	0.0018 (7)	−0.0059 (7)	−0.0036 (6)
C3	0.0599 (9)	0.0475 (8)	0.0472 (8)	0.0040 (7)	−0.0039 (7)	0.0018 (7)
C4	0.0451 (7)	0.0393 (7)	0.0505 (8)	−0.0025 (5)	0.0082 (6)	−0.0028 (6)
C5	0.0603 (9)	0.0493 (8)	0.0469 (9)	−0.0030 (7)	−0.0046 (7)	−0.0075 (7)
C6	0.0619 (9)	0.0460 (8)	0.0467 (8)	0.0008 (7)	−0.0074 (7)	0.0005 (7)
C7	0.0531 (8)	0.0422 (8)	0.0487 (8)	−0.0052 (6)	0.0022 (6)	−0.0018 (6)
C8	0.0674 (11)	0.0448 (9)	0.0731 (13)	0.0038 (8)	−0.0067 (10)	−0.0086 (8)

### Geometric parameters (Å, º)

**Table:**

O1—C7	1.2286 (19)	C1—C6	1.393 (2)
O2—N3	1.2363 (18)	C2—C3	1.385 (2)
O3—N3	1.2284 (19)	C2—H1	0.96 (2)
O4—N3	1.2314 (18)	C3—C4	1.372 (2)
O1W—H1W	0.93 (3)	C3—H2	0.93 (2)
O1W—H2W	0.78 (3)	C4—C5	1.375 (2)
N1—C7	1.349 (2)	C5—C6	1.380 (2)
N1—C1	1.4129 (19)	C5—H8	0.91 (2)
N1—H3	0.91 (2)	C6—H7	0.96 (2)
N2—C4	1.4698 (19)	C7—C8	1.495 (2)
N2—H4	0.93 (2)	C8—H9	0.93 (4)
N2—H5	0.89 (3)	C8—H10	0.93 (3)
N2—H6	0.93 (3)	C8—H11	0.86 (4)
C1—C2	1.387 (2)		
			
H1W—O1W—H2W	103 (2)	C4—C3—H2	117.5 (13)
C7—N1—C1	128.48 (14)	C2—C3—H2	122.6 (13)
C7—N1—H3	117.0 (12)	C3—C4—C5	120.93 (14)
C1—N1—H3	114.4 (12)	C3—C4—N2	119.78 (14)
C4—N2—H4	111.1 (13)	C5—C4—N2	119.29 (14)
C4—N2—H5	107.7 (19)	C4—C5—C6	119.47 (15)
H4—N2—H5	115 (2)	C4—C5—H8	123.0 (13)
C4—N2—H6	109.9 (16)	C6—C5—H8	117.5 (13)
H4—N2—H6	97.5 (19)	C5—C6—C1	120.52 (15)
H5—N2—H6	116 (2)	C5—C6—H7	120.4 (12)
O3—N3—O4	121.44 (15)	C1—C6—H7	119.0 (12)
O3—N3—O2	120.58 (15)	O1—C7—N1	122.92 (14)
O4—N3—O2	117.98 (14)	O1—C7—C8	121.34 (15)
C2—C1—C6	119.12 (14)	N1—C7—C8	115.75 (15)
C2—C1—N1	124.34 (14)	C7—C8—H9	115 (2)
C6—C1—N1	116.53 (13)	C7—C8—H10	114.1 (18)
C3—C2—C1	120.07 (15)	H9—C8—H10	106 (3)
C3—C2—H1	117.5 (11)	C7—C8—H11	110 (2)
C1—C2—H1	122.4 (11)	H9—C8—H11	107 (3)
C4—C3—C2	119.89 (15)	H10—C8—H11	104 (3)

### Hydrogen-bond geometry (Å, º)

**Table:**

*D*—H···*A*	*D*—H	H···*A*	*D*···*A*	*D*—H···*A*
O1*W*—H1*W*···O1	0.93 (3)	1.79 (3)	2.689 (2)	161 (3)
O1*W*—H2*W*···O3	0.78 (3)	2.03 (3)	2.805 (2)	172 (3)
N1—H3···O2^i^	0.91 (2)	2.24 (2)	3.117 (2)	161 (2)
N2—H4···O1*W*^ii^	0.93 (2)	1.87 (2)	2.796 (2)	173 (2)
N2—H5···O2^iii^	0.89 (3)	2.14 (3)	3.008 (2)	165 (3)
N2—H6···O1*W*^iv^	0.93 (3)	1.95 (2)	2.827 (2)	156 (2)

Symmetry codes: (i) *x*−1, −*y*+1/2, *z*−1/2; (ii) −*x*+1, −*y*, −*z*+1; (iii) −*x*+1, *y*−1/2, −*z*+1/2; (iv) −*x*, −*y*, −*z*+1.
